# *The Plant Cell* is accepting applications for assistant features editors for 2024

**DOI:** 10.1093/plcell/koad171

**Published:** 2023-06-21

**Authors:** Nancy A Eckardt, Blake C Meyers

**Affiliations:** Senior Features Editor, The Plant Cell, American Society of Plant Biologists, USA; Editor-in-Chief, The Plant Cell, American Society of Plant Biologists, USA; Donald Danforth Plant Science Center, St. Louis, MO 63132, USA; Division of Plant Sciences and Technology, University of Missouri-Columbia, Columbia, MO 65211, USA

Are you a postdoc or senior graduate student who loves writing, reading, and communicating the hottest new research in plant science? We invite you to apply to *The Plant Cell* Assistant Features Editor (AFE) program for 2024. Our AFEs write In Brief articles for *The Plant Cell* to highlight new research published in the journal, gain experience and training in writing for a broad audience, receive training in the peer review process, and enjoy networking opportunities with members of our editorial board.

*The Plant Cell* AFE program is highly competitive and a great stepping stone to a successful career in many fields of plant science. Skills gained in science writing and networking opportunities as an AFE (with journal editors, authors, and readers) will serve you well in a future career as an independent researcher in academia or industry, other careers in industry or non-governmental organizations, or in scientific publishing.

AFEs are expected to contribute a minimum of three In Brief articles per year, highlighting recent articles in their areas of interest, and to participate in editing the work of their fellow AFEs. In addition to gaining experience in science writing, editing, and publishing in *The Plant Cell*, AFEs join our editorial board and receive training in the peer review process through participation in editorial board meetings and consultations. The 2024 AFEs will be provided with financial support to attend *The Plant Cell* Editorial board meeting and ASPB centennial celebration at Plant Biology 2024 in Honolulu, Hawaii, in June 2024.

We are accepting applications through Monday, September 18, 2023, for new AFEs for 2024. Preference will be given to postdoctoral researchers with a proven track record of excellence in research, writing, and a commitment to communicating science. Details on how to apply can be found in the advertisement at https://plantae.org/blog/call-for-tpc-afes/. Applicants are asked to submit a cover letter, CV, letters of recommendation from two referees, and two writing samples: a first-author paper and a sample In Brief article highlighting a research article in *The Plant Cell* selected from the list provided in the advertisement.

